# If Cumulative Risk Assessment Is the Answer, What Is the Question?

**DOI:** 10.1289/ehp.9330

**Published:** 2007-01-24

**Authors:** Michael A. Callahan, Ken Sexton

**Affiliations:** 1 U.S. Environmental Protection Agency, Region 6, Dallas, Texas, USA; 2 University of Texas School of Public Health, Brownsville Regional Campus, Brownsville, Texas, USA

**Keywords:** additivity assumption, combined risk, cumulative risk, mixtures, multiple stressors, risk assessment guidelines

## Abstract

Cumulative risk refers to the combined threats from exposure via all relevant routes to multiple stressors including biological, chemical, physical, and psychosocial entities. Cumulative risk assessment is a tool for organizing and analyzing information to examine, characterize, and possibly quantify the combined adverse effects on human health or ecologic resources from multiple environmental stressors. The U.S. Environmental Protection Agency (EPA) has initiated a long-term effort to develop future guidelines for cumulative risk assessment, including publication in 2003 of a framework that describes important features of the process and discusses theoretical issues, technical matters, and key definitions. The framework divides the process of cumulative risk assessment into three interrelated phases: *a*) planning, scoping, and problem formulation; *b*) analysis; and *c*) interpretation and risk characterization. It also discusses the additional complexities introduced by attempts to analyze cumulative risks from multiple stressors and describes some of the theoretical approaches that can be used. The development of guidelines for cumulative risk assessment is an essential element in the transition of the U.S. EPA risk assessment methodology from a narrow focus on a single stressor, end point, source, pathway, and exposure route to a broader, more holistic approach involving analysis of combined effects of cumulative exposure to multiple stressors via all relevant sources, pathways, and routes.

## Introduction

Risk is a socially constructed and culturally mediated concept (Jasanoff 1991; Kasperson and Kasperson 1991; Linder 1997) that is used to give meaning to things, forces, or circumstances that pose danger to people or what they value [National Research Council (NRC) 1996]. Various forms of risk assessment have been around for centuries (Bernstein 1997) and each society has its own particular hazards that are of special concern (Kasperson and Kasperson 1991). During the latter half of the 20th century, risk from deleterious by-products of economic activity and technology came to be seen by industrialized societies as a noxious quality present in varying degrees in different environmental settings and geographic locations. Although risk is not necessarily an intrinsically quantifiable variable, virtually all of the formalized assessment methods that subsequently evolved, including those at the U.S. Environmental Protection Agency (EPA), implicitly assume that risk can be estimated, measured or expressed in numerical terms. Today, a quantitative, or at least semiquantitative, description of severity and likelihood of harm is the dominant paradigm for expressing risk from environmental hazards (NRC 1983, 1994, 1996).

### Historical perspective

During the mid-1970s the U.S. Food and Drug Administration (U.S. FDA) and the U.S. EPA began to adopt systematic methods for assessing human health risks from exposure to environmental carcinogens. By the early 1980s, risk assessment played an important role in many regulatory decisions, and there were individuals in the public and private sectors who identified themselves as “risk assessors.” The Supreme Court’s 1980 decision in *Industrial Union Department, AFL-CIO v. American Petroleum Institute*, 448 U.S. 607 [cited in NRC (1994)], also known as the “Benzene Decision,” provided a major push for development of risk assessment within regulatory agencies. The decision struck down the benezene standard developed by the Occupational Safety and Health Administration (OSHA), which was based on the policy of trying to reduce carcinogens in the workplace as far as technologically possible without consideration of whether actual concentrations posed a significant health risk. The court found that OSHA could regulate under the Occupational Safety and Health Act only if it determined that benzene posed a significant risk of harm, thus sending a strong signal that quantitative risk assessment was necessary prior to decisions about which risks justified regulatory intervention (NRC 1994).

In 1983 the NRC issued its landmark report “Risk Assessment in the Federal Government: Managing the Process,” also known as the “Red Book” (NRC 1983). This report provided a synthesis of relevant concepts and scientific principles and recommendations of specific methods for the conduct of risk assessment. One major recommendation was that regulatory agencies develop and use guidelines that specify the scientific basis for the conduct of risk assessment and that establish default options.

The U.S. EPA was the only federal regulatory agency to follow this recommendation (NRC 1994), publishing a set of guidelines for carcinogen risk assessment in 1986 (U.S. EPA 1986a). These and subsequent guidelines set forth recommended principles and procedures to guide U.S. EPA scientists in assessing the risk from chemicals or other agents in the environment and to inform decision makers and stakeholders about these procedures. A series of risk assessment guidelines were ultimately published, including guidelines for carcinogenicity (U.S. EPA 1976, 1986a, 2005a, 2005b), chemical mixtures (U.S. EPA 1986b, 2000), mutagenicity (U.S. EPA 1986c), developmental toxicity (U.S. EPA 1991), exposure assessment (U.S. EPA 1992a), reproductive toxicity (U.S. EPA 1996), neurotoxicity (U.S. EPA 1998a), and ecologic risk (U.S. EPA 1998b).

The risk assessment approach that evolved at the U.S. EPA had its roots in the pressing regulatory issues of the 1970s and 1980s, such as carcinogenic air and water pollution from heavy industry (Albert et al. 1977; NRC 1983). Traditional risk assessment was strongly influenced by the regulatory mind-set of that earlier time, which emphasized national command-and-control strategies and technology-based regulations to control pollution on a chemical-by-chemical basis. Although most analysts acknowledge that substantial progress was made in reducing pollution from the largest and most obvious sources, by the 1990s more complicated and nuanced problems were attracting regulatory attention, and questions of trade-offs between regulatory costs and benefits to society were gaining prominence [National Academy of Public Administration (NAPA) 1995; NRC 1994, 1996; U.S. EPA 1990, 1992b]. Many argued that the existing environmental management system needed serious overhaul to meet the complex challenges of the 21st century, such as global climate change and endocrine-disrupting chemicals (Breyer 1993; Howard 1994; NAPA 1995; Sexton et al. 1999). At the same time, there were calls for related changes in the risk assessment process to bring it into line with new strategic directions and priorities [Browner 1995; NRC 1994, 1996; The Presidential/Congressional Commission on Risk Assessment and Risk Management (PCCRARM 1997); Sexton 1997; U.S. EPA 1995].

One of the most prominent risk-related issues presently confronting regulatory decision makers is the need to evaluate combined (cumulative) risk to human populations and ecologic resources from concurrent exposure to multiple environmental stressors. Although researchers and risk assessors have recognized the need to address this problem since at least the 1970s, progress has been slow because of insufficient knowledge, inadequate understanding, technologic limitations, and scarce funding (Carpenter et al. 2002; Monosson 2005; Sexton et al. 1995). Despite the many obstacles that need to be overcome, the U.S. EPA has initiated a long-term effort aimed at developing guidelines for cumulative risk assessment (U.S. EPA 1997a, 2003). In this article, we briefly *a*) examine the scientific and risk assessment background for this effort, *b*) describe the U.S. EPA’s framework for cumulative risk assessment, and *c*) discuss the framework in the context of a larger transition in the approach of the U.S. EPA to risk assessment and risk management.

## Scientific and Risk Assessment Background

It is a well-established principle in toxicology that simultaneous or sequential exposure to two or more environmental agents can modify the consequences of exposure to those agents acting alone (Ottoboni 1984). Among the better known examples of interactive effects are increased combined risks of lung cancer from exposure to tobacco smoke and asbestos (Erren et al. 1999) or radon (Morrison et al. 1998), and increased risk of hepatocellular carcinoma from exposure to aflatoxin and hepatitis B infection (Kuper et al. 2001). In ecologic risk, modified consequences from interactive effects of multiple stressors are sometimes called “cascading impacts.”

Studying the combined effects of mixtures is difficult whether it involves toxicologic experiments with laboratory animals, field studies of contaminated habitats, or epidemiologic investigations of naturally occurring populations. For example, although toxicity studies of chemical mixtures have been conducted in laboratories for decades, most with simple binary mixtures, the study of more complex mixtures that mimic real-world conditions is problematic. Conducting a relatively straightforward factorial design that examines the interactions of three chemicals at five different dose levels with, for example, 6 animals per group, requires 125 treatment groups. The cost of studying these 750 laboratory animals is substantial, yet the knowledge gained applies only to one temporal sequence of exposures at one postexposure time point (Suk et al. 2002). Similarly, although epidemiologic studies have proved useful for studying many chemical mixtures, such as cigarette smoke and diesel exhaust, they usually involve large and expensive field operations, and errors in exposure estimation can weaken epidemiologic evidence substantially (Samet 1995).

Over the past decade, research on adverse effects from exposure to environmental stressor mixtures, particularly chemical mixtures, has increased substantially (Carpenter et al. 2002; Feron et al. 2002; Monosson 2005; Seed et al. 1995; Suk and Olden 2004). Although the results have expanded our knowledge base, in most cases realistic risk assessment is hindered by a scarcity of data on the combined effects of exposure to real-world mixtures. The U.S. EPA is, nevertheless, faced with public demands for action on issues such as childhood pesticide exposures (NRC 1993; Wargo 1998) and endocrine-disrupting chemicals (Colborn et al. 1997) and is driven by statutory requirements, such as the Food Quality Protection Act of 1996 (FQPA), that direct the agency to consider cumulative risks as part of regulatory decisions to protect public health (FQPA 1996). To assist risk assessors, the U.S. EPA published “Supplementary Guidance for Conducting Health Risk Assessment of Chemical Mixtures” (U.S. EPA 2000), which updates its 1986 chemical mixture guidelines (U.S. EPA 1986a).

The U.S. EPA guidance states that use of mixture-specific toxicity data is the preferred method for characterizing cumulative risks and is most appropriate for fairly consistent mixtures such as environmental tobacco smoke, diesel exhaust, commercial pesticide formulations, and coke oven emissions. Typically, however, toxicity data on the mixtures of regulatory interest are not available, in which case, the U.S. EPA recommends combining toxicity information for each individual chemical in an additive manner unless there is convincing data to the contrary. The U.S. EPA suggests that information on potential interactions among mixture components be incorporated into the assessment when it is available, but in the absence of such data, additivity of dose or response is assumed to be the default condition (U.S. EPA 2000).

### Additivity assumption

Dose addition, which assumes that the toxicity of individual chemicals in the mixture can be calculated relative to each other or to a common chemical, is recommended for compounds that have the same mechanism of toxicity or that damage the same target organ. The U.S. EPA identifies three methods for dose addition: relative potency factors (RPFs), toxic equivalency factors (TEFs), and the hazard index (HI). When mechanisms of action are relatively well characterized, the U.S. EPA suggests using either RPFs or TEFs. In the RPF approach, the toxicity of each chemical in the mixture is scaled by its relative potency compared to an index chemical, which is toxicologically well characterized and representative of other chemicals in the mixture. The TEF approach is a special case of the RPF method, and uses “extensive mechanistic information that shows all toxic effects of concern share a common mode of action” to determine relative potencies (U.S. EPA 2000). The RPF method has been used to assess cumulative risk of organophosphate pesticides and *N*-methyl carbamate (U.S. EPA 2002a, 2005c), and the TEF approach has been used for mixtures of organochlorine compounds such as dioxins and dioxin-like polychlorinated biphenyls (van den Berg et al. 1998; Walker et al. 2005).

If little or no mechanistic data are available, the U.S. EPA recommends using the HI to assess cumulative risks for chemicals that have an established chronic reference dose (RfD) or reference concentration (RfC). The RfD (which addresses exposure by ingestion or dermal contact) or the RfC (which addresses exposure by inhalation) is the dose or concentration to which an individual can be exposed over a lifetime with a reasonable certainty of no harm. In the HI approach, the exposure concentration of each chemical in the mixture is divided by its RfD or RfC to calculate a ”hazard quotient” (HQ). These HQs are then added together to calculate the HI for the whole mixture. A mixture with an HI ≤ 1 is interpreted to mean that the corresponding exposure is unlikely to be harmful, whereas a value > 1 suggests that further toxicologic and mechanistic evaluations may be needed (U.S. EPA 2000). One primary application of the HI approach has been the assessment of combined risks from exposure to hazardous air pollutants (Caldwell et al. 1998; Fox et al. 2004; Morello-Frosch et al. 2000; Tam and Neumann 2004).

For ecologic risk assessments on hazardous waste, the U.S. EPA places additional restrictions on using the hazard index (U.S. EPA 1997b). The same toxicity mechanism and/or target organ must be demonstrated before HQs can be added to generate an HI. An HI can only be calculated for groups of chemicals having the same toxic mechanism. Further, the guidance (U.S. EPA 1997b) stipulates that the RfDs or RfCs used to calculate the HI must be for the same exposure duration category (i.e., chronic, subchronic, or acute).

One example of a human health HI with applicability to cumulative risk assessments is the interaction-based HI method, which incorporates binary interaction data to modify the HI. It assumes that two-way interactions among various chemicals account for most of the mixture interactions and, therefore, can collectively describe the combined effects of the mixture of interest. The interaction-based HI procedure also involves an analysis of the weight-of-the-evidence related to the nature of the chemical interactions, the plausibility that interactions will occur, and the relevance of the interactions for human health (U.S. EPA 2000).

Response addition, like dose addition, assumes no component interactions and is recommended by the U.S. EPA when chemicals in a mixture act independently or have a different critical end point so that the presence of one chemical does not affect the toxicity of another. Historically, the application of this method has focused primarily on chemical carcinogens (U.S. EPA 2000).

### Selecting a method

The universal default option underpinning U.S. EPA’s guidance for assessment of cumulative risk from chemical mixtures is the assumption (in the absence of data to the contrary) that doses and/or responses are additive. The “additivity assumption” provides a convenient and practical postulation that allows risk assessors to make a rough calculation, which U.S. EPA believes represents a reasonable and neutral risk estimate (U.S. EPA 2000). But in most cases a lack of scientific knowledge and mechanistic understanding precludes determination of whether the calculated value is an overestimate (e.g., because of antagonistic interactions) or an underestimate (e.g., because of synergistic interactions) of the actual risk.

The choice of methods for conducting a cumulative risk assessment for chemicals acting by a common mechanism depends on both the objectives of the analysis and the limitations of the available data. Depending on the circumstances, application of more than one method may be justified, and a simple, less data-intensive method may be appropriate for initial screening, whereas a more intricate and data-intensive method might be appropriate for follow-up analysis. Data requirements generally increase as one moves from basic HI methods to RPF and TEF methods to interactive HI methods. The most realistic assessments are those that use biologically based cumulative risk models to incorporate important toxicokinetic and toxicodynamic parameters into final risk estimates. Unfortunately, these approaches also require the most data on toxicologic interactions among mixture constituents, and in virtually all cases, construction or application of biologically based cumulative risk models is stymied by data deficiencies (Mileson et al. 1999; Sexton and Hattis 2007).

In addition to the chemical mixture guidance described above, the U.S. EPA has undertaken numerous activities related to cumulative risk assessment. For example, the U.S. EPA National Center for Environmental Assessment has published ecologic risk assessment guidelines (U.S. EPA 1998b) that incorporate cumulative risk considerations, and five watershed case studies have been prepared that demonstrate the methods. The Office of Air and Radiation has performed assessments of cumulative risks from hazardous air pollutants, and the Office of Pesticide Programs has developed guidance for conducting cumulative risk assessments of pesticides (U.S. EPA 2002b). The Superfund Program has included some evaluation of cumulative effects from chemicals in its guidance on risk assessments (U.S. EPA, 1989), and several U.S. EPA regional offices have carried out cumulative risk projects (U.S. EPA 2003).

## Framework for Cumulative Risk Assessment

In May 2003 the U.S. EPA published its “Framework for Cumulative Risk Assessment” (known as the “Framework”) to serve as a foundation for development of future guidelines that promote consistency across U.S. EPA offices and programs. Building on the U.S. EPA’s growing experience, it provides a conceptual framework to identify the fundamental elements and basic principles of an organized process for conducting and evaluating assessments of cumulative risk. It also offers a flexible structure that encourages dialogue on theoretical issues, technical matters, key definitions, and implementation issues. Overall, the Framework is an information document that describes important features of cumulative risk assessment “whether or not the methods or data currently exist to adequately analyze or evaluate those aspects of the assessment” (U.S. EPA 2003).

In the Framework, “cumulative risk” is defined as the combined risks from aggregate exposure (i.e., including all relevant routes) to multiple agents or stressors, including biological (e.g., *Mycobacterium tuberculosis*), chemical (e.g., toluene), physical (e.g., noise), and psychosocial (e.g., job- or family-related stress) entities. The term “cumulative risk assessment” is defined as an analysis, characterization, and possible quantification of the combined risks to human health or the environment from multiple agents or stressors (U.S. EPA 2003).

Cumulative human health risk assessment is distinct from traditional U.S. EPA human health risk assessments in four ways. First, cumulative risk assessment does not necessarily have to be quantitative; a qualitative analysis may be appropriate depending on the circumstances. Second, the combined effects of more than one agent or stressor are assessed, as opposed to the individual effects of single agents or stressors that have historically been the focus of most risk assessments. Third, attention is shifted from conventional source-based assessments of hypothetical individuals to population-based assessments of “real” individuals or populations that are potentially affected by the combined stressors of interest. Fourth, evaluation of cumulative risk broadens the spectrum of environmental agents and stressors being assessed beyond the traditional, nearly exclusive focus on chemicals (U.S. EPA 2003).

In contrast to human health risk assessments, U.S. EPA’s ecologic risk assessments (U.S. EPA 1998b) tend to be qualitative or only semiquantitative. In some cases, the complexity of ecologic systems necessitates that assessment of combined effects be conducted, as when toxicity tests are conducted on contaminated sediments. Moreover, ecologic risk assessments generally focus on “real” or relevant receptors, which may include biotic populations or communities. The reality is that there is as much room for improvement and refinement of cumulative ecologic risk assessment as there is for cumulative human health risk assessment.

**Figure 1:**
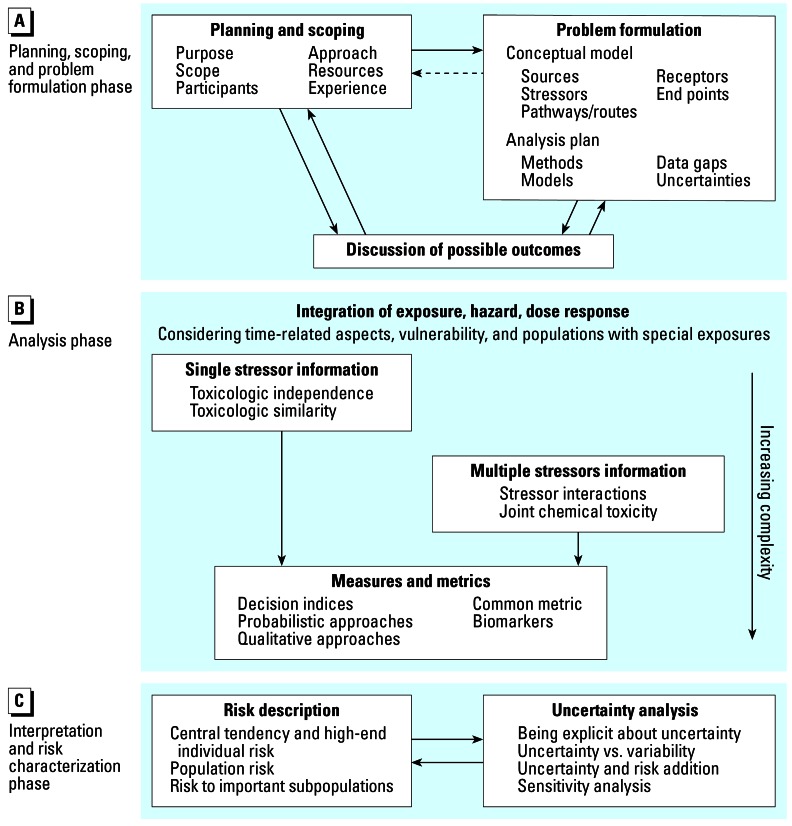
The three interactive phases of cumulative risk assessment [adapted from U.S. EPA (2003)].

The Framework, as shown in Figure 1, describes three interrelated and generally sequential phases for cumulative risk assessment: *A*) planning, scoping, and problem formulation; *B*) analysis; and *C*) interpretation and risk characterization. In the first phase, a team of risk assessors, risk managers, and interested stakeholders work together to determine the goals, breadth, depth, and focus of the assessment. The products of the planning, scoping, and problem formulation phase are *a*) a conceptual model that identifies the stressors to be evaluated, the health or environmental effects to be evaluated, and the relationships among various exposures and effects, and *b*) an analysis plan that specifies the data needed, the approach to be taken, and the types of results expected during the subsequent phase (U.S. EPA 2003).

The analysis phase involves developing exposure profiles, examining the nature and extent of interactions among stressors, estimating risks to the population(s) of interest, and discussing related variability and uncertainty. Among the difficult technical issues that need to be addressed and resolved during this phase are the description of interactions among stressors and their effects on mixture toxicity, estimation of cumulative exposure to the stressors of interest, and identification of vulnerable groups. The product of the analysis phase is an estimate of the combined risks of exposure to multiple stressors for the population(s) of interest, along with an estimate of the uncertainty and variability associated with this estimate (U.S. EPA 2003).

In the final phase—interpretation and risk characterization—the risk estimates are explained and put into perspective in terms of their significance, their reliability, and the overall confidence placed in them. In addition, the effects of key assumptions on final risk estimates are described, the uncertainties involved are delineated, and a determination is made as to whether the assessment met the goals and objectives set forth in phase one (U.S. EPA 2003).

### Increased complexity

Assessing combined effects, including the potential for antagonistic and synergistic interactions, among diverse mixture constituents that may include biological, chemical, physical, and psychosocial stressors is substantially more complex methodologically and computationally than traditional single-chemical, source-oriented assessments (deFur et al. 2007; Menzie et al. 2007; Ryan et al. 2007; Sexton and Hattis 2007). Although a few examples of cumulative risk assessments attempt to evaluate joint effects of a variety of different kinds of stressors (Barnthouse et al. 2000), in most cases the underlying scientific uncertainties, technical challenges, and methodologic complications have discouraged extensive application of these approaches. To illustrate the increased complexity of cumulative risk assessments compared with single-stressor risk assessments, four mixture-related challenges must be addressed: consideration of the time-related aspects of exposure; determination of the vulnerability of exposed groups and populations; identification of subpopulations with exposures of special concern; and characterization of interactions between psychosocial stress and other factors (U.S. EPA 2003).

### Time-related aspects of exposure

Conventional risk assessments typically assume that adverse effects are related to a combination of exposure intensity and duration. The U.S. EPA assumes, for example, that cancer risk is proportional to lifetime dose. But there are cases where the details and sequence of exposure may be important for predicting risk, particularly for multiple stressors. For example, past exposure to one stressor may predispose an individual or population to be more vulnerable to subsequent exposure to another stressor (e.g., Durkin et al. 1995). It is important, therefore, that exposure data supporting a cumulative risk assessment (e.g., co-occurrence with other stressors, continuous versus intermittent exposure, simultaneous versus sequential contact) be collected to conserve the covariance and dependency that exists among the stressors of interest (U.S. EPA 2003).

### Vulnerability

The vulnerability of a human population or ecosystem has been defined as “the capacity to be wounded from a perturbation or stress, whether environmental or socioeconomic, upon peoples, systems, or other receptors” (Kasperson and Kasperson 2001). Cumulative risk assessment is a tool that can be useful for evaluating one or more of the four basic types of vulnerability: biological susceptibility to adverse effects of stressors (e.g., based on such factors as genetic predilection, age, sex, health status, differential sensitivities of ecologic species, and life stages); differential exposure to multiple stressors (e.g., greater cumulative body burden); differential preparedness to withstand stressor effects (e.g., immunization in humans, previous acclimation, and genetic drift in ecologic species); and differential ability to recover from stressor effects (e.g., access to health care in humans, ability to leave the contaminated area, and differential fecundities in ecologic species). However, before cumulative risk assessment can be effective in this regard, much work is needed to establish relationships between the different types of vulnerability factors and changes in human and ecologic risk (U.S. EPA 2003).

### At-risk populations

The process of identifying subpopulations (or ecologic populations) that may experience higher-than-average exposures is more complicated in a cumulative risk assessment because we are concerned with combined exposures to multiple stressors via all relevant routes, pathways, and sources (Ryan et al. 2007; Sexton and Hattis 2007). Examples of potentially at-risk groups are those exposed either directly or indirectly to occupational stressors; those living, working, or playing in proximity to major sources of stressors; and those with activity patterns or lifestyles that bring them into contact with stressors. Because traditional exposure assessments have tended to focus on single chemicals, single routes of exposures, and specific sources or source categories, methods for cumulative exposure assessment are not well developed and appropriate data are rarely available (Sexton and Hattis 2007; U.S. EPA 2003).

### Psychosocial stress

Cumulative risk assessment explicitly acknowledges the importance of assessing the effects of nontraditional factors such as psychosocial stress from family conflict, poverty, underemployment, unemployment, unsafe working environment, discrimination, residential crowding, inadequate housing quality, street crime, traffic congestion, and dilapidated neighborhood conditions. Although there is ample evidence that stress can induce or reveal a latent effect of certain toxicants or that it can alter basal levels of biological functioning and shift toxicity thresholds, methods and techniques for assessing levels of stress and their potential contributions to cumulative risk are in their infancy (deFur et al. 2007). Similarly, in ecologic systems, very few studies have been conducted on toxicant effects induced by contributing stress factors such as habitat fragmentation and alteration. Thus, in most cases, risk assessors do not have the necessary tools to evaluate interactions among these factors adequately (deFur et al. 2007; Menzie et al. 2007; U.S. EPA 2003).

Nonetheless, the importance of including psychosocial stress in cumulative risk assessments can be demonstrated using an anecdotal example based on real events. Suppose monitoring data show that toxic chemicals from a Superfund site have contaminated a nearby stream that an Indian tribe has used for generations as a tribal fishing ground. The concentrations of several chemicals are determined to be above health-related benchmarks, causing state regulatory officials to close the stream to all fishing and to issue health advisories asking people not to eat fish from the stream until further notice. From the narrow perspective of traditional risk assessment, the problem is solved because if no fish are being consumed, there is no exposure and, therefore, no risk of related adverse effects (e.g., cancer).

From the tribal standpoint, however, members are forced to choose between continuing long standing (sometimes sacred) traditions and cultural practices and protecting the health and safety of their children. The result is substantial stress within and among families, leading to disagreements among members of the tribe about how to respond. Eventually, the psychologic and social stress of choosing between two “unacceptable” choices leads to strife and fragmentation within the tribe, generating yet more stress and uncertainty.

But these harmful “cascading effects” resulting from the initial contamination were not considered in the risk-based decision about how to deal with the polluted stream. The reality is that these types of stressors and effects are not often considered as part of traditional risk assessments, either quantitatively or qualitatively. As a result, the tribe concluded that risk assessment “did not work for them” because it ignored a major environmentally induced effect on tribal members—the consequent stress and resulting community fragmentation that started with the polluted stream.

It is this “narrowness” of conventional risk assessment that has spawned skepticism among many community members, both tribal and nontribal, about the use of traditional risk-based decisions. Cumulative risk assessment is meant to broaden the scope and relevance of the analysis by explicitly including evaluation of important factors such as psychosocial stress, even if quantitative methods are not available.

### Theoretical approaches

There are several different theoretical approaches for predicting risk from exposure to multiple stressors (U.S. EPA 2003). For example, the joint exposure–response relationship for a mixture of stressors can be approximated using only information on individual stressors if one assumes either toxicologic independence or toxicologic similarity. In the case of toxicologic independence, single stressor data are sufficient to estimate the joint exposure-response linkage as long as the toxicity modes of action are biologically independent and there are no pretoxicity interactions (e.g., metabolic inhibition). For toxicologic similarity, stressors are grouped according to a common mode of action for each adverse effect of concern, then for all effects caused by a particular mode of action, the assumption of dose addition can be applied to the stressors in that group (e.g., relative potency factors, toxic equivalency factors).

Simplifying assumptions regarding exposure (e.g., all exposures occur continuously, the sequence of exposures is unimportant, mixture composition is constant over time) and dose-response (e.g., one dose–response curve can serve as a “bounding estimate”) allows for the dose–response evaluation to occur separately from the exposure assessment step. This method can be used to set health-protective action levels by estimating upper bounds on toxic potency and exposure and lower bounds on the acceptable exposure level. However, large uncertainties may be introduced if the simplifying assumptions are not valid or if dose–response conditions do not represent the same conditions as the exposure scenario.

Cumulative risk assessments often require combining divergent data from a variety of sources. For example, exposure data for some stressors may be expressed as time-weighted averages, whereas for others, continuous data may be available. Similarly, toxicity data may allow estimation of probabilistic risk for some stressors, while providing only qualitative descriptions for others. In these kinds of situations, decision indices can be used where appropriate to convert dissimilar multivariate data into a single number (U.S. EPA 2003). The most common example used for cumulative health risk assessment is the HI for a specific chemical mixture. Although each HI is specific for a single target organ, it usually reflects numerous studies of individual mixture constituents that often involve multiple species of laboratory animals and a range of exposure levels. The main disadvantages of a decision index approach such as this are that the uncertainties in the calculation are largely hidden, there is no agreed-upon way to quantify a risk if the index exceeds the decision threshold (i.e., HI value > 1), and the method frequently involves quantifying scientific judgments.

Probabilistic approaches to cumulative risk assessment may be appropriate in certain situations, but careful consideration must be given to defining the set of relevant end points because it has important logistical and practical implications for calculating and interpreting risk. Probabilistic approaches are facilitated by defining the risk of a given end point in terms of population risk, such as the predicted number of cases for a particular end point. It can also be helpful to define the risk of a particular end point in respect to only those individuals who are at the high end of the exposure distribution (e.g., living at the fence line of a point source) or to those individuals who will incur the greatest increased risk (e.g., children who are more biologically susceptible because of age or size). The use of multichemical, multipathway, probabilistic approaches for cumulative risk assessment has been illustrated in assessments conducted for several pesticide groups (U.S. EPA, 2002a, 2005c).

Finally, it is likely, at least initially, that there will be many cases where cumulative risk cannot be quantified in any meaningful or reliable way. Qualitative approaches may be the only practical means to overcome the problems of complexity and data deficiencies and provide some insight into the nature and magnitude of cumulative risks (e.g., in the tribal example discussed earlier). Broad indicators, such as indication of high, medium, or low for various factors in the assessment, might be used to communicate complicated and disparate data related to exposure (e.g., emission inventories, environmental concentrations) and toxicity [such as toxicity indicators displayed using geographic information systems (GIS)]. Geographically based measures of hazard, such as GIS maps displaying data on the release locations and toxicity of chemicals, are potentially useful indicators of possible exposures to environmental mixtures and might serve as “direction finders” for identifying likely “hot spots” or at-risk populations. Although qualitative results may be converted to semiquantitative findings (e.g., assigning numerical scores to scientific judgments about high, medium, and low cumulative risks), and they can be used as supplementary material for quantitative assessments (for example, by adding a descriptive appendix), in some instances it may be neither feasible nor desirable to quantify cumulative risks. Overall, it is important to bear in mind that qualitative assessments of cumulative risk have value in and of themselves.

## Risk Assessment in Transition

The U.S. EPA’s efforts to institutionalize and standardize procedures for cumulative risk assessment are occurring as part of a larger transition in the way the agency assesses and manages environmental risks. By the mid-1990s, many inside (Browner 1995; U.S. EPA 1995, 1997a) and outside (NAPA 1995; NRC 1994, 1996; PCCRARM 1997; Sexton 1997) the U.S. EPA had come to believe that conventional risk assessment needed to be revamped to make it more relevant to the problems confronting decision makers. The U.S. EPA was increasingly faced with the need to move beyond the agency’s early focus on command-and-control strategies, end-of-pipe controls, narrow media-based statutes, one-size-fits-all regulations, rigid and prescriptive rules, and process-based “best technology” standards. To meet the complex challenges of the new millennium, many argued that the U.S. EPA needed to concentrate more on cooperative and voluntary strategies, pollution prevention, holistic multi-media approaches, place-based environmental decisions, flexible and easy-to-adjust rules, and outcome-based standards (Breyer 1993; Howard 1994; NAPA 1995; Sexton 2006; Sexton et al. 1999).

These sorts of changes in regulatory philosophy and approach required complementary changes in risk assessment principles and practices (Browner 1995; NRC 1994, 1996; U.S. EPA 1995, 1997a). Today, U.S. EPA’s risk assessment emphasis is shifting away from a narrow focus on single stressors, end points, sources, pathways, and environmental media to a more expansive application that gives prominence to multiple stressors, end points, sources, pathways, and environmental media (Table 1). This ongoing transition has proceeded unevenly, propelled at various times by recommendations from the National Academy of Sciences (Institute of Medicine 1999; NRC 1993, 1994, 1996), public calls for environmental justice (Bryant and Mohai 1992; Bullard 1994), Executive Orders (Clinton 1994), and Congressional fiat (FQPA 1996).

The U.S. EPA has officially embraced and encouraged the transition, starting in 1995 when Carol M. Browner, then U.S. EPA Administrator, issued the following statement (Browner 1995).

. . . the challenges we face now are very different from those of the past. . . . If we are to succeed and build our credibility and stature as a leader in environmental protection in the next century, EPA must be responsive and resolve to more openly and fully communicate to the public the complexities and challenges of environmental decision making in the face of scientific uncertainty. . . . we must improve the way in which we characterize and communicate environmental risk. . . . While I believe that the American public expects us to err on the side of protection in the face of scientific uncertainty, I do not want our assessments to be unrealistically conservative. We cannot lead the fight for environmental protection into the next century unless we use common sense in all we do.

In 1997 the U.S. EPA Science Policy Council (U.S. EPA 1997a) continued:

The practice of risk assessment within the Environmental Protection Agency (EPA) is evolving away from a focus on the potential of a single pollutant in one environmental medium for causing cancer toward integrated assessments involving suites of pollutants in several media that may cause a variety of adverse effects on humans, plants, animals, or even effects on ecological systems and their processes and functions. . . . The scope of Agency risk assessments describes the current identifiable context of the environmental risk that will (or can) be analyzed. It is defined according to *who* or *what* is at risk of *adverse effects* from identifiable *sources* and *stressors* through several *routes of exposure* over varied time frames.

The importance of cumulative risk as both a catalyst and a cornerstone for the risk assessment transition portrayed in Table 1 was brought home in 2003 with publication of the U.S. EPA’s “Framework for Cumulative Risk Assessment” (U.S. EPA 2003), which stated:

Cumulative risk assessments will identify the need for many different kinds of data – some of them are not the data commonly now used for risk assessment – and often, cumulative risk assessment will demand large quantities of such data. . . . As of August 1, 2001, there were 19,533 pesticide products on the market, and 79,120 existing chemicals on the TSCA inventory. Each year, an additional number of chemicals are added. Assessing the cumulative effect of these chemicals will be a great challenge to the Agency and may be the primary issue in the risk assessment field in the next ten years.

## Conclusions

Human populations and ecologic systems are commonly exposed to a diverse and dynamic mixture of biological, chemical, physical, and psychosocial stressors as part of their everyday existence. Conventional risk assessment methodology, when it has addressed this issue at all, has relied on simplifying assumptions, both implicit and explicit, about combined effects from exposure to environmental mixtures. In general, these simplifying assumptions, such as evaluating the risks of chemicals separately and adding resultant risks, or incorporation of a 3- to 10-fold uncertainty factor for interindividual variability into many of the RfDs and RfCs, are meant to foster conservative (protective) risk estimates. But data are rarely available to determine the validity of resulting approximations. Moreover, when the questions to be addressed by the risk assessment involve evaluating the joint probabilities of harm from a number of stressors, both chemical and nonchemical, the boundary conditions leading to the simplifying assumptions of conventional risk assessment often break down. It is these kinds of questions that demand the conceptual approaches and evaluation methods of cumulative risk assessment.

Cumulative risk assessment is a tool for organizing and analyzing information about combined effects of exposure to multiple environmental stressors. Consequently, it can provide more realistic answers to the kinds of critical environmental questions that are increasingly being asked by a wide spectrum of society, including affected populations, environmental groups, business organizations, legislators, and academics. Examples of pressing real-world questions include the following. Are residents of poor inner-city neighborhoods at higher-than-average risk from environmental stressors? What are the risks to vulnerable ecosystems from the combined effects of urban sprawl? Do combinations of endocrine-disrupting chemicals pose a significant risk to humans or wildlife? If numerous industrial facilities are located in a certain area, even though each is at or below its statutory emission limits, do cumulative risks from these aggregate emissions still pose potential harm to nearby residents?

Conventional risk assessment has not been able to address these sorts of questions effectively, often leading to stakeholder disenchantment with both the process and its products. In the past, for example, risk assessment has been lampooned as a series of unsubstantiated assumptions compounded by rash speculation. It has also been compared unfavorably with meteorologic forecasting, as when it is said that the only difference between a 5-year weather forecast and a risk assessment is that in 5 years you will know whether the weather forecast was right. In the end, however, regardless of all its faults and shortcomings, most would agree with the sentiment expressed by Winston Churchill (Churchill 1947) in describing democracy, and modified here to make it apropos for risk assessment—“formalized risk assessment is the worst method for analyzing environmental harm, except for all the others.”

Cumulative risk assessment is an essential element in the transition of the U.S. EPA risk methodology from a concentration on single (primarily chemical) stressors, end points, sources, pathways, and routes of exposure to a broader, more holistic approach involving analysis of combined effects of cumulative exposure to multiple (not necessarily just chemical) stressors via all relevant sources, pathways, and routes. This change may not satisfy all the critics, especially in the short-term, as many of the methods are still being developed, but eventually it will go a long way toward making risk assessment more reliable, realistic, and relevant.

## Figures and Tables

**Table 1:** Comparison of risk assessment and risk management characteristics for the traditional versus the emerging approach at the U.S. EPA.

Traditional risk assessment and management characteristics	Emerging risk assessment and management characteristics
Single end point	Multiple end points
Single source	Multiple sources
Single pathway	Multiple pathways
Single route of exposure	Multiple routes of exposure
Single-media focus	Multimedia focus
Single-stressor risk reduction	Multistressor risk reduction
Centralized decision making	Community-based decision making
Command-and-control strategies	Flexibility in achieving goals
One-size-fits-all responses	Case-specific responses

Adapted from U.S. EPA (1997a).
